# Effect of maternal serum magnesium and calcium levels on umbilical glial fibrillary acidic protein levels in preterm labor

**DOI:** 10.1038/s41598-023-40022-x

**Published:** 2023-08-16

**Authors:** Dhanny Primantara Johari Santoso, Annisa Dewi Nugrahani, Amillia Siddiq, Akhmad Yogi Pramatirta, Muhammad Alamsyah Aziz, Setyorini Irianti, Adhi Pribadi, Anita Deborah Anwar, Jusuf Sulaeman Effendi

**Affiliations:** 1 Maternal-Fetal Medicine Division, Department of Obstetrics and Gynaecology, Slamet General District Hospital Garut, Faculty of Medicine, Padjadjaran University – Dr. Hasan Sadikin General Hospital, Pasteur No. 38, Bandung, 40161 West Java Indonesia; 2 Maternal-Fetal Medicine Division, Department of Obstetrics and Gynaecology, Faculty of Medicine, Padjadjaran University – Dr. Hasan Sadikin General Hospital, Bandung, Indonesia

**Keywords:** Reproductive disorders, Neurophysiology, Predictive markers

## Abstract

Magnesium can prevent astrocyte cell death and Glial Fibrillary Acidic Protein (GFAP) secretion as inflammatory marker in preterm delivery. This study was performed to analyze differences in umbilical cord GFAP levels in preterm labor given magnesium sulfate (MgSO_4_) as treatment group and control group and analyze the correlation between magnesium and calcium levels with umbilical GFAP levels. This quasi-experimental study was performed on 68 patients at Dr. Hasan Sadikin General Hospital from February-June 2021 consisting of 34 patients in each group. Maternal-umbilical cord magnesium levels, calcium levels, and GFAP levels were examined using ELISA test. The result was statistically measured by IBM SPSS 24.0. We found that there was a significant difference between maternal and umbilical magnesium levels and GFAP umbilical cord blood levels between the treatment and the control group (*P* < 0.05) in which GFAP level was higher in the control group. The multivariate analysis showed no significant relevance between mother magnesium and calcium level to umbilical cord GFAP level in the MgSO_4_ group. As conclusions, umbilical cord blood GFAP levels in preterm labor given MgSO_4_ were lower than in preterm deliveries who were not given MgSO_4_. There was no correlation between magnesium, calcium, and GFAP levels in the treatment group.

## Introduction

The incidence of preterm labor is still high even in modern countries and still shows an increasing trend. Indonesia is in the top five countries dealing with premature babies, consisting of 675,700 premature babies per year. The incidence of cerebral palsy shows an increase of 2–2.5 per 1000 preterm deliveries. The risk of cerebral palsy is higher in premature babies. As a consequence, low quality of human resources may become new problems in the future^1–3^.

In preterm pregnancy, there is an inflammatory reaction in the fetal brain that will trigger the activity of inflammatory factors. The presence of immature oligodendrocyte cells will trigger the hyperactivity of microglia and astrocytes, causing damage to astrocyte cells in the brain and Glial Fibrillary Acidic Protein (GFAP) secretion as a specific marker in the brain. Hypoxia and ischemia at the cellular level triggered by the anaerobic metabolism of glutamate and lactate can rapidly lead to cell apoptosis and cause brain neuronal cell death^1,4–7^.

Definitive mechanisms of antenatal administration of MgSO_4_ are as fetal neuroprotector are preventing tissue from free radicals and hypoxic damage. Magnesium promotes neurogenesis in premature brain cell maturation by stimulating neurotrophic factor secretion. Hypoxia and inflammation will cause the membrane pathway of the hemichannels to open, which can also be due to oxygen and glucose deprivation, metabolic inhibition, or low levels of extracellular calcium ions (Ca^2+^). Magnesium is an endogenous calcium antagonist and supports the blockade of N-methyl D-aspartate receptors (NMDAr), which is highly dependent on the surrounding ionic tension^1,4–7^. Magnesium also prevents the secondary cascade of astrocytes that lead to cell death. Magnesium also reduces the excitotoxicity damage induced by glutamate agonists. Extracellular glutamate will decrease on magnesium administration after focal cerebral ischemia. It will increase the survival of primary oligodendrocyte precursor cells. However, long-term administration of magnesium alone tends to cause hypocalcemia^1,5–11^.

Giving magnesium nutrition and its relationship with blood calcium level followed by GFAP examination can be used to predict the risk of preterm labor morbidity. The aim of this study to analyze the differences of umbilical cord blood GFAP levels in preterm labor given MgSO_4_ and those not and the relationship between magnesium and calcium levels of pregnant women with umbilical GFAP levels.

## Materials and methods

### Study design research subjects

This experimental study aimed to find the relationship between magnesium levels in pregnant women and calcium levels in pregnant women, and levels of Glial Fibrillary Acidic Protein (GFAP) in preterm labor 28^+0^–34^+6^ weeks from February-June 2021. We use the protective effect of MgSO_4_ until 34 weeks of gestation, given the size of the protective effect in the previous systematic review and study protocol (MAGENTA study protocol), the ongoing uncertainty about benefits at later gestational ages, and the serious health and financial consequences of cerebral palsy for the child, family, and society, a magnesium sulphate trial for women at risk of preterm birth between 30 and 34 weeks' gestation is both significant and pertinent for clinical practice globally^12^.

Participants were included if they met the inclusion and exclusion criteria. All methods were carried out in accordance with relevant guidelines and regulations after obtaining approval and recommendations from the Ethics Committee Review Board of Hasan Sadikin General Hospital – Faculty of Medicine, Universitas Padjadjaran with reference number LB.02.01/X.6.5.176/2021. This study was conducted according to Declaration of Helsinki. Written informed consent was obtained from all patients. Patients were provided with written and verbal information about the study.

### Inclusion criteria

Preterm labor patients beyond 17 years and between 28^+0^ and 34^+6^ gestational weeks in Obstetrics and Gynaecology Department, Hasan Sadikin General Hospital Bandung were included in this study. They had been given consent to participate in the study by filling out an informed consent form. In the treatment group, the patients were included when there were indications that the birth process would begin in the following 12 h or those who had entered the opening of the latent stage of the active phase. Latency of the patients was considered if there was no parturient after 8 h of loading dose and maintenance dose of MgSO_4_ *(descriebd in data collection methods, section duration of treatment in the treatment group).* Patients with preterm labor and ruptured membranes were also included because they were also imminent to preterm labor.

### Exclusion criteria

Exclusion criteria were consisted of patients with previous complication caused by MgSO_4_ treatment (> 24 h), refusal to participate, and emergent adverse event during the study.

### Data collection methods

A total of 68 preterm deliveries that met the study criteria were divided into two groups (treatment and control groups). All samples were collected using consecutive samplings.

- Duration of Treatment in The Treatment Group: The initial dose of four grams of MgSO_4_ was given in 100 mL of NaCl and finished in 15 min. Then the maintenance dose of ten grams of MgSO_4_ in 500 mL of Ringer Lactate was administered at a dose of 1 g/hour. Blood samples of the treatment group were taken an hour after the initial administration of MgSO_4_.
- Treatment of Control Group: The MgSO_4_ was not given in control group because they were already in > 8 cm dilation or they were expected to enter labor process in less than two hours. In comparison to treatment group, the control group blood samples were taken when the mother initially came.

Laboratory examinations of maternal magnesium levels, maternal calcium levels, umbilical cord magnesium levels, umbilical cord calcium levels, and umbilical vein GFAP levels were carried out with ELISA (Enzyme-linked immunosorbent assay). The sample was allowed to settle for two hours at room temperature or left overnight at 4° before being centrifuged for 15 min at 1000xg and 2–8 °C. The supernatant was taken for examination of GFAP levels. If the inspection is not carried out immediately, the supernatant must be stored at − 4 °C (seven days) or − 21 °C (a month).

### Statistical analysis

The results of the examination were recorded and analyzed. The analysis were performed using the IBM SPSS program for windows 24.0 using Mann–Whitney, Pearson, and Spearman test as well as multivariate analysis.

## Results

### Subject characteristics

The following data of each participant was retrieved: maternal age, parity, gestational age, and body mass index (Table 1). The statistical tests on the variables of age, gestational age, BMI, and the number of gravida were not statistically significant (*P* > 0.05). There was no difference in the proportion of statistically significant variables of age, gestational age, BMI, and the number of gravida in both groups.

**Table 1:** Comparison of the characteristics of pregnant women between the treatment group and the control group.

Variable	Group		*P*-value
	MgSO_4_	Non MgSO_4_	
	N = 34	N = 34	
Maternal Age (years)			0.966
Mean ± Std	27.62 ± 5.057	28.41 ± 7.955	
Median	29.00	26.00	
Range (min–max)	18.00–35.00	17.00–44.00	
Gestational age (weeks)			0.723
Mean ± Std	31.79 ± 1.591	31.56 ± 1.894	
Median	32.00	32.00	
Range (min–max)	28.00–34.00	27.00–34.00	
Gravidae			0.430
Mean ± Std	1.85 ± 1.077	2.24 ± 1.499	
Median	1.50	2.00	
Range (min–max)	1.00–5.00	1.00–6.00	
BMI (kg/m^2^)			0.717
Mean ± Std	26.59 ± 5.223	25.27 ± 3.804	
Median	25.33	25.60	
Range (min–max)	18.67–35.60	16.44–32.05	

For numerical data, the *p*-value is tested by unpaired T-test if the data is normally distributed with the alternative of Mann Whitney test if the data is not normally distributed. The value of significance is based on the value of *p* < 0.05. The * sign indicates the value of *p* < 0.05, which means that it is statistically significant or significant. * Significance < 0.05, ** significance < 0.01.

### Comparison of magnesium and calcium levels as well as umbilical GFAP levels MgSO_4_ treatment vs non treatment groups

In this study, there was a significant mean increase in the treatment group following MgSO_4_ administration. Various studies have shown lower serum maternal magnesium levels compared to non-pregnant women^13^ and lower magnesium levels in women with preterm labor than in full-term delivery^14–16^.

The differences between maternal magnesium levels and umbilical cord magnesium levels were statistically significant in both groups (P < 0.05). Then, the differences in GFAP levels between both groups were also significant (P < 0.05). The mean maternal serum magnesium level in the treatment group was higher compared to the control group (Table 2). However, it was still within normal limit (1.58–6.32 mg/dl or 0.65–2.6 mmol/L). Therefore, there was no unexpected hypermagnesemia effect, such as nausea, vomiting, hypotension, tachycardia, respiratory distress, and pulmonary oedema. Magnesium levels in the treatment group were similar to MgSO_4_ levels in the study with the administration of MgSO_4_ as neuroprotector, which is 2.43–3.16 mg/dl^16–20^.

**Table 2:** Comparison of magnesium and calcium levels as well as umbilical GFAP levels in both groups.

Variable	Group		*P*-value
	MgSO_4_	Non MgSO_4_	
	N = 34	N = 34	
Maternal Magnesium Levels (mg/dL)			0.0001**
Mean ± Std (mg/dl)	3.18 ± 0.553	1.90 ± 0.605	
Median	3.20	1.74	
Range (min–max)	1.89–4.30	1.30–4.13	
Maternal Calcium Levels (mg/dL)			0.568
Mean ± Std (mg/dL)	8.08 ± 0.565	8.16 ± 0.535	
Median	8.10	8.20	
Range (min–max)	6.60–9.10	6.60–9.10	
Umbilical Magnesium Levels (mg/dL)			0.0001**
Mean ± Std	3.10 ± 0.641	2.19 ± 0.716	
Median	3.12	1.95	
Range (min–max)	1.99–4.39	1.67–5.18	
Umbilical Calcium Levels (mg/dL)			0.680
Mean ± Std	9.69 ± 1.050	9.58 ± 1.579	
Median	9.55	9.85	
Range (min–max)	6.20–12.30	2.00–11.70	
Umbilical GFAP Levels(mg/dL)			0.0001**
Mean ± Std	0.03 ± 0.012	0.05 ± 0.020	
Median	0.03	0.04	
Range (min–max)	0.01–0.07	0.02–0.11	

For numerical data, the p-value is tested by unpaired T-test if the data is normally distributed with the alternative of Mann Whitney test if the data is not normally distributed. The value of significance is based on the value of *p* < 0.05. The * sign indicates the value of *p* < 0.05, which means that it is statistically significant or significant. * Significance < 0.05, ** significance < 0.01, units in mg/dl.

The level of umbilical magnesium in the treatment group in this study was significantly higher than in the control group. The previous studies showed that magnesium had been absorbed from the mother to the placenta and umbilical cord, with the ratio of maternal serum and umbilical magnesium in the MgSO_4_ group reaching 0.97, similar to other studies 0.94-1^16,21,22^.

GFAP levels in this study are described in Table 2 as numerical data. There was a significant difference in GFAP levels umbilical cord in both groups. The GFAP value in the group treated with MgSO_4_ was lower than in the non-MgSO_4_ group (control group).

### Detailed comparison of umbilical GFAP levels in the treatment group and control group

GFAP is a specific protein released by the brain, especially astrocytes, in response to trauma, so its value is expected to increase in brain injury and remain low in the absence of brain injury^23–26^. Table 3 explains that in the group given magnesium sulfate, there were 28 samples below the reagent detection limit issued by the factory that issued the reagent, which was below 0.045 ng/l. This result showed that the group given MgSO_4_ had several samples with low GFAP values compared to the group that was not given MgSO_4_. There was a statistically significant difference in the proportion between the outcome variables in the group given MgSO_4_ and the group who was not given MgSO_4_ treatment (control group).

**Table 3:** Comparison of umbilical GFAP levels in the treatment group and control group.

Variable	Group		*P* Value
	MgSO4	Non MgSO4	
	N = 34	N = 34	
Umbilical GFAP levels			0.010*
Results below detection limit	28(82.4%)	18(52.9%)	
Extrapolated results	6(17.6%)	16(47.1%)	

Categorical data *p*-value is calculated based on the Chi-Square test with the alternative Kolmogorov Smirnov test and Fisher's Exact if the requirements of the Chi-Square are not met. The significance value is based on the *p*-value < 0.05. statistically meaningful. * Significance < 0.05, ** significance < 0.01, detection limit value 0.045.

### Correlation analysis of maternal magnesium levels, maternal calcium levels, umbilical magnesium levels, and umbilical calcium levels with GFAP levels in the MgSO_4_ group

Following Table 4, there was a moderate correlation or relationship with a negative and significant correlation direction between GFAP levels and umbilical cord calcium levels in the group given MgSO_4_. From the Pearson correlation test results, it could be concluded that there is a minimal and negligible correlation or relationship with a negative and insignificant correlation direction between GFAP levels and magnesium levels, calcium levels, and umbilical cord magnesium levels in the group given MgSO_4_.

**Table 4:** Correlation analysis of all variables in MgSO_4_ (treatment) group.

Variable	Correlation	R	*P*-*Value*
Correlation of maternal magnesium levels with GFAP levels	*Pearson*	− 0.118	0.507
Correlation of maternal calcium levels with GFAP levels	*Pearson*	− 0.088	0.619
Correlation of umbilical magnesium levels with GFAP levels	*Pearson*	− 0.009	0.961
Correlation of umbilical calcium levels with GFAP levels	*Spearman*	− 0.454	0.007*

The significance value of *p* < 0.05. The sign ** indicates statistically significant or significant. r: correlation coefficient.

## Discussions

The age of participants included was beyond 17 years to reduce the risk factors for preterm delivery and the risk of grade 3 and 4 intraventricular haemorrhage in neonates at maternal age below 17 years^8^. All study subjects with 28^+0^ to 34^+6^ weeks of gestation age were taken to limit prognostic factors of poorer neurologic development at < 28 weeks of gestational age^9,10^. In addition, the use of antenatal MgSO_4_ as a neuroprotector at < 28 weeks of gestation is still limited in studies conducted in developed countries^11^.

Various studies have shown lower serum maternal magnesium levels compared to non-pregnant women^13^ and lower magnesium levels in women with preterm labor than in full-term delivery^14–16^. Several studies demonstrate an increase in maternal magnesium levels during preterm compared to term deliveries. Hypoxia and ischemia at the cellular level triggered by the anaerobic metabolism of glutamate and lactate due to preterm labor can rapidly lead to cell apoptosis and cause brain neuronal cell death^1,4–7^.In this study, there was a significant mean increase in the treatment group following MgSO_4_ administration.

The administration of magnesium as a neuroprotector aims to increase serum magnesium levels in the umbilical cord blood, which will also affect calcium levels in the umbilical cord. Massive neuroprotective therapy and prolonged duration of administration were correlated with depression of serum calcium levels^26–30^. Several studies also stated that there was no significant relationship between calcium levels and the incidence of hypoxemic-ischemic encephalopathy. However, there is a low serum magnesium level in the incidence of severe asphyxia and hypoxemic-ischemic encephalopathy^27–32^. In preterm labor, hypoxemic ischemic encephalopathy can occur, which can cause perinatal complications^32^. Neuroprotective management is initiated at the time of imminent preterm delivery to prevent long-term sequelae^29^.

There were no maternal life-threatening side effects in the study using the MgSO_4_ study, such as respiratory failure, cardiac arrest, changes in heart function, and death^33^. However, minor side effects were expected, such as redness, headache, feeling warm, sweating, nausea, and vomiting, and disappeared with symptomatic management. According to Cochrane 2012, there was no substantial difference in terms of side effects in mothers who were given MgSO_4_ as a neuroprotector^34–37^.

Magnesium has been proven as a neuroprotector in various studies with suspected mechanisms through various pathways. There are as an endogenous calcium antagonist, preventing excitotoxic damage through NDMAr blockade, and magnesium's role in downregulation the inflammatory cascade^27^. It appears to have a similar competitive function in blocking intracellular Ca^2+^ channels, decreasing calcium availability, and preventing smooth muscle contractility. In addition, magnesium also competes with calcium at the motor endplate, decreasing excitation by preventing the excess release of acetylcholine^26,38^. In this study, the relationship between maternal magnesium, maternal calcium, and umbilical cord GFAP levels did not provide a significant difference. The correlation of maternal magnesium, maternal calcium, and GFAP levels is not significant in this study and could indicate some conjecture. This finding could be postulated to occur since the examination of umbilical cord GFAP levels was carried out on the first day after birth, while the evaluation should be done on the third day and should be confirmed by neuroimaging^39^. However, due to restrictions imposed by local regulations, neonates without substantial clinical symptoms were sent home without confirmation by a neuroimaging study on the first postpartum day. This could have masked the association between maternal magnesium, maternal calcium, and umbilical cord GFAP levels,

This study tries to proof that GFAP levels in preterm labor given MgSO_4_ were lower than in preterm deliveries who were not given MgSO_4_. Therefore, this finding could form a basis of further hypothesis that GFAP could have a role as a biomarker of nerve cell injury, although very few studies discuss about this topic. However, GFAP is not the only parameter. The administration of magnesium as a neuroprotector in preterm labor has had many studies trying to prove it. However, the scientific pathway of being a neuroprotector at the level of communication signals between cells and receptors is multifactorial^34–37^.

These studies did not distinguish the history of magnesium sulfate administration as a tocolytic and as a confounding factor. Various mechanisms of magnesium as a neuroprotector in various pathways should be further investigated to obtain intermediate biomarkers. Another major limitation was that there weren't enough women to draw any conclusive results about BMI, gestational week, single or multiple births, intrauterine growth restriction, or blood flow restriction in the umbilical artery. However, as novelties found in this study, magnesium supplementation and its relationship with blood calcium level followed by GFAP examination can be used to predict the risk of preterm labor morbidity. Further research can be in a more significant number of samples or initial calcium data before treatment.

## Conclusions

Umbilical GFAP levels in preterm labor given MgSO_4_ were lower than in preterm labor not given MgSO_4_. This finding could form a basis of further hypothesis that GFAP could have a role as a biomarker of nerve cell injury in preterm labor while MgSO_4_ could be as neuroprotector in preterm labor. Moreover, there was no relationship between maternal serum magnesium levels, maternal serum calcium levels with umbilical GFAP levels in the group given MgSO_4_ that could indicate some conjecture including proper timing and methods of testing for nerve injury in neonates. These findings could become the basis and opportunities for further more comprehensive researches.

## Data Availability

The datasets used and/or analyzed during the current study are available from the corresponding author upon reasonable request.

## Abbreviations

BMI: Body mass index
Ca^2+^: Calcium ions
ELISA: Enzyme-linked immunosorbent assay
GFAP: Glial fibrillary acidic protein
MgSO_4_: Magnesium sulfate
NMDAr: N-methyl D-aspartate receptors
